# Supplementary material for: Transient inhibition of 53BP1 increases the frequency of targeted integration in human hematopoietic stem and progenitor cells
Source: Nat Commun. 2024 Jan 2;15:111. doi: 10.1038/s41467-023-43413-w (PMC10762240; doi:10.1038/s41467-023-43413-w)
Supplement: Supplementary file 1 — Supplementary Information [file 41467_2023_43413_MOESM1_ESM.pdf]

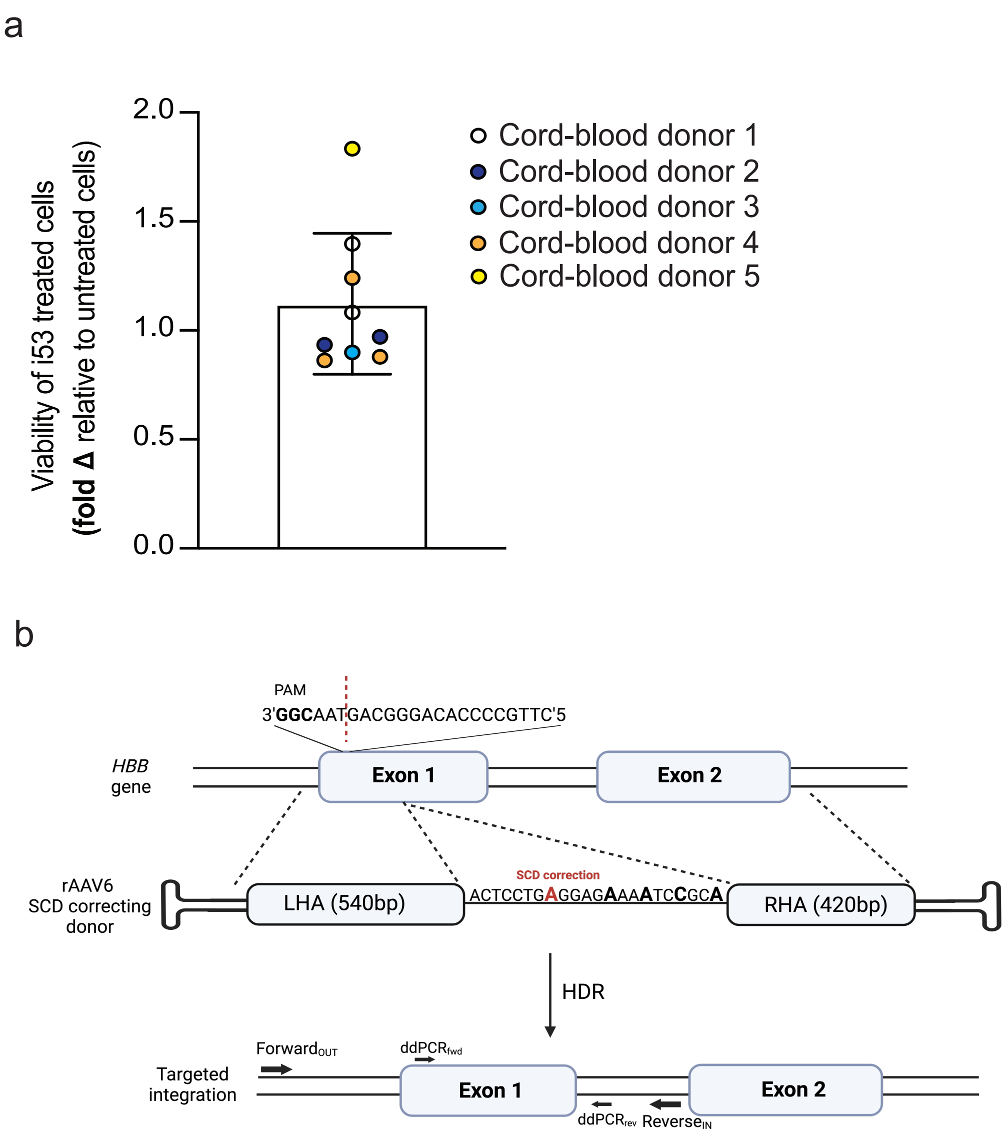

Supplementary Figure 1. Optimizing i53 peptide for targeting *HBB* locus in CD34⁺ HSPCs. (a) Viability of i53 treated (5000µg/ml) cells relative to untreated cells. Data from n=5 biological donors. Mean±SD depicted. **(b)** Schematic of the rAAV6 SCD correcting donor with LHA and RHA represented. sgRNA used for *HBB* gene and SCD correction sequences, and ddPCR primers are all depicted (figure created with Biorender.com).

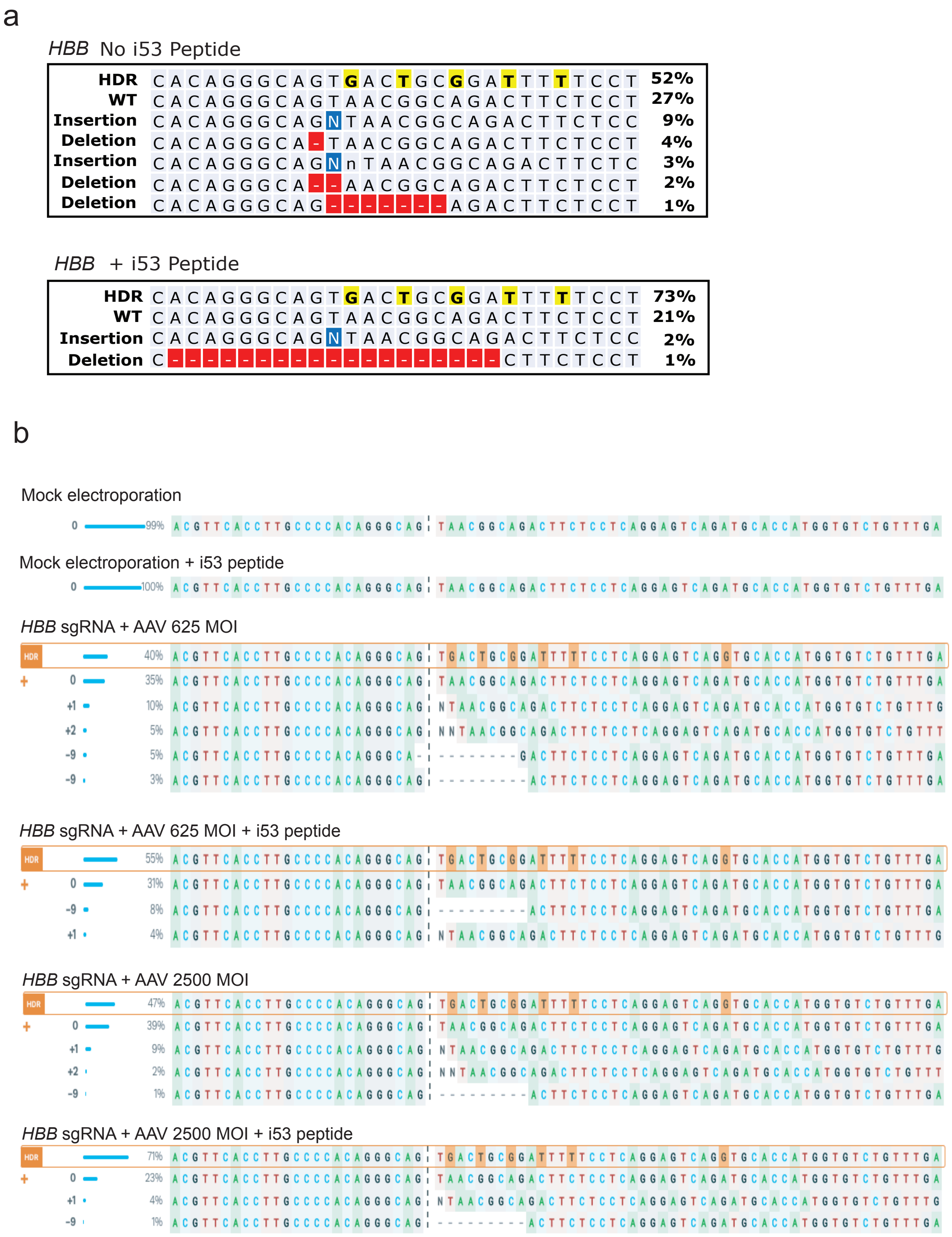

Supplementary Figure S2. Allelic outcomes at *HBB* locus. (a) Allele spectra and corresponding percentages of alleles generated following editing with *HBB* targeting sgRNA+ RNP with or without i53 peptide. **(b)** Additional allele spectra obtained from ICE analysis and corresponding percentages of alleles generated following editing with *HBB* targeting sgRNA+ RNP + 625 MOI AAV6 or 2500 MOI AAV6 with or without i53 peptide.

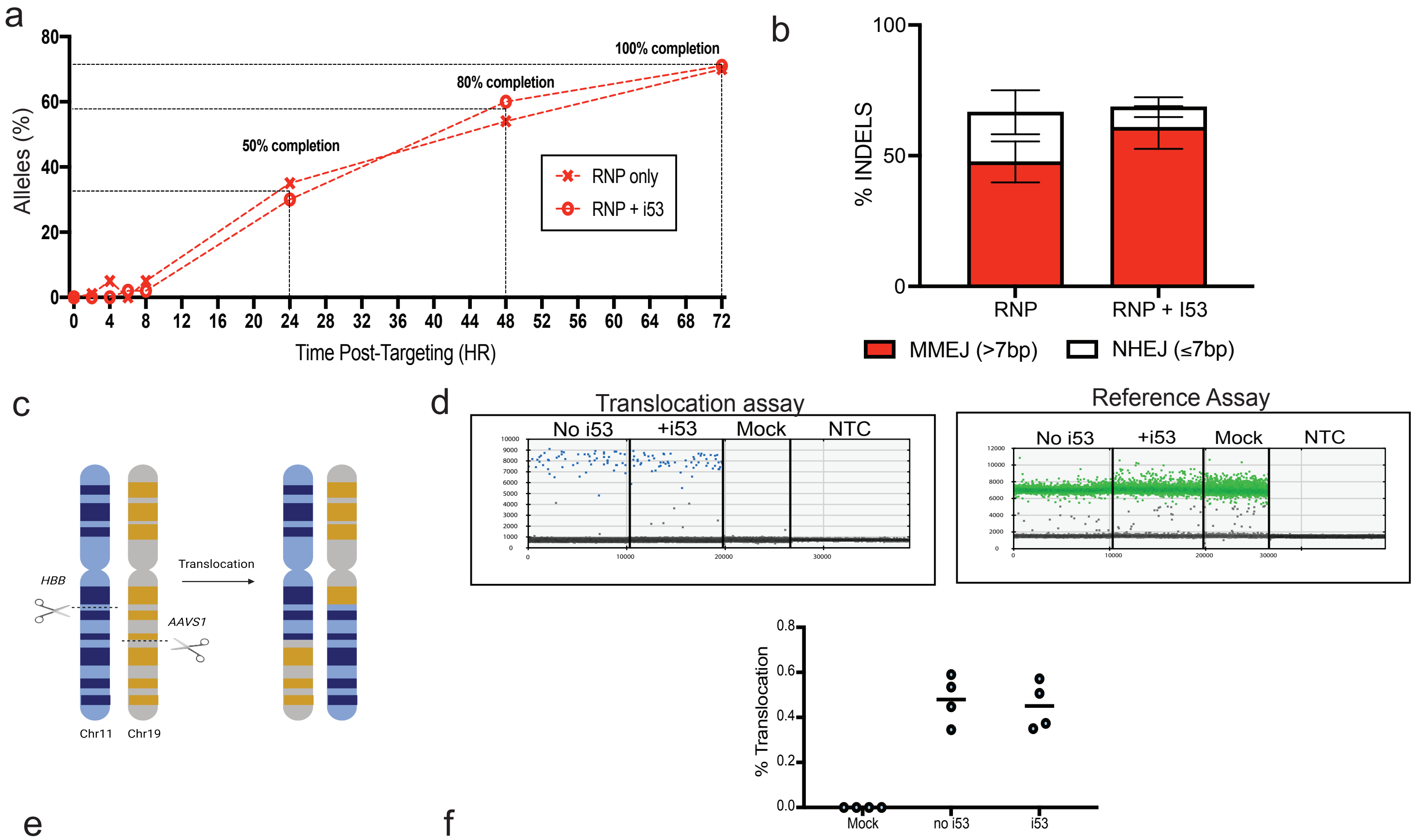

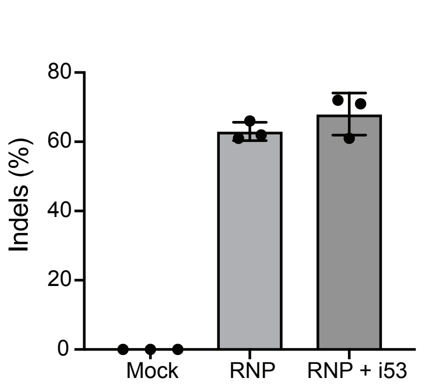

f

mPB Donor 1

ON	start	end	reads	hits
chr11	5211405	5240754	2633824	12656

OMT	start	end	reads	hits
*chr9	101833338	101833846	968	6

NBS	start	end	reads	hits
chr11	797505	798023	2925	12

*This OMT was only significant in one of the replicates

mPB Donor 1 + i53

ON	start	end	reads	hits
chr11	5216033	5240756	3874364	14356

OMT	start	end	reads	hits
chr9	101833339	101833850	8001	21

NBS	start	end	reads	hits
chr11	5211402	5214101	6586	18
chr11	5257472	5258565	2023	8

mPB Donor 2

ON	start	end	reads	hits
chr11	5211655	5239637	1875852	9927

NBS	start	end	reads	hits
chr11	5241552	5248361	3106	19
chr11	5192902	5196859	1051	17

mPB Donor 2 + i53

ON	start	end	reads	hits
chr11	5209695	5244743	2167658	11417

NBS	start	end	reads	hits
chr11	5192902	5196504	4826	37
chr11	5177049	5178795	3046	13
chr11	5271427	5272828	1004	4

Supplementary Figure 3. Additional translocation assay to show safety of i53 treatment (a) and **(b)** Indel frequencies measured in HSPCs edited with RNP or RNP + i53 peptide in the absence of AAV donor. **(a)** Data from n=2 biological donors, each donor conducted with technical duplicates, Mean value reported. **(b)** Data from n=3 biological replicates. Mean±SD depicted. **(c)** Schematic showing the *HBB* gene on chromosome 11 and the *AAVS1* locus on chromosome 19. The Cas9 cut sites are shown in dashed line. Possible monocentric translocation shown. Created with BioRender.com **(d)** Representative ddPCR analyses quantifying translocations in NTC (non-template control), mock-electroporated, and RNP edited and RNP+i53 edited cells. The reference assay quantifies TERT gene copies used to normalize for DNA input. Quantification of % translocation shown. Data from n=4 biological donors. **(e)** Indel frequencies were assessed by ddPCR. Data from n=3 biological replicates. **(f)** CAST-Seq results for *HBB* targeting with or without i53 in CD34+ HSPCs. Listed are on-target aberrations, OMTs, and NBS for all samples. Data from n=3 biological replicates. Mean±SD depicted.

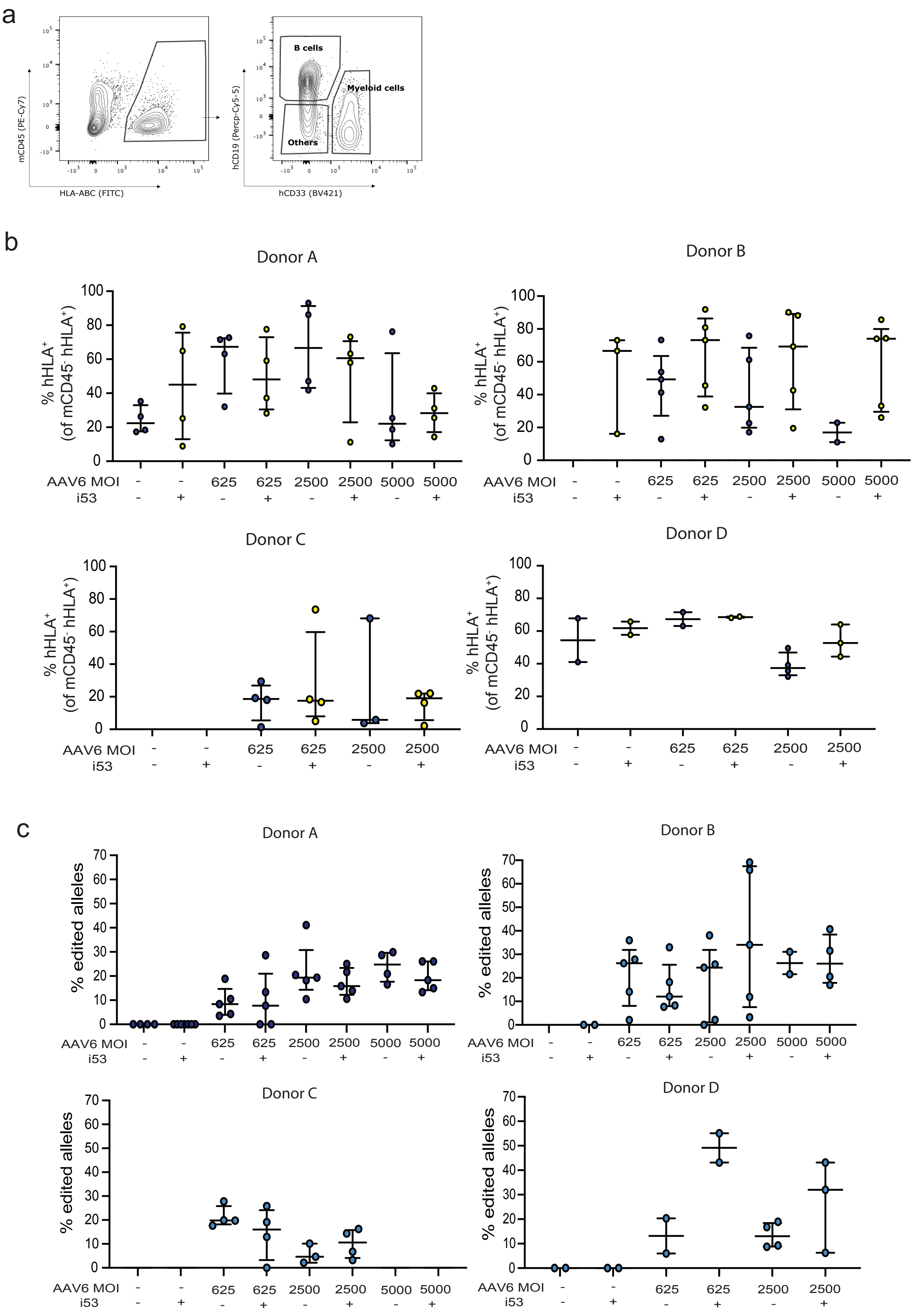
a
mCD45 (PE-Cy7)
HLA-ABC (FITC)
B cells
Myeloid cells
Others
hCD19 (Percp-Cy5-5)
hCD33 (BV421)
b
Donor A
Donor B
Donor C
Donor D
% hHLA+ (of mCD45- hHLA+)
AAV6 MOI
i53
625
2500
5000
c
% edited alleles

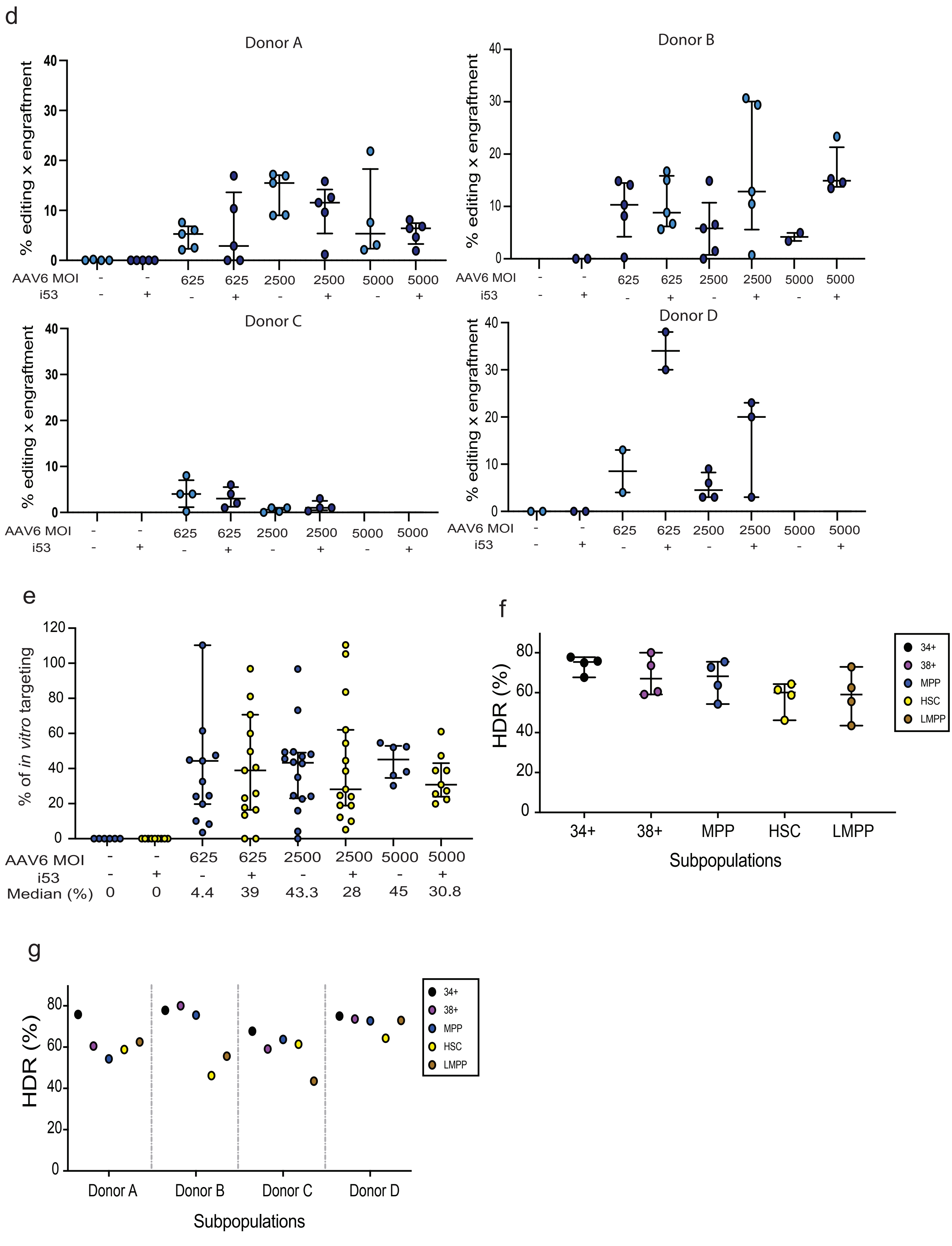
d
Donor A
Donor B
Donor C
Donor D
% editing x engraftment
AAV6 MOI
i53
- - 625 625 2500 2500 5000 5000
- + - + - + - +
e
% of in vitro targeting
AAV6 MOI
i53
Median (%)
0 0 4.4 39 43.3 28 45 30.8
f
HDR (%)
34+
38+
MPP
HSC
LMPP
Subpopulations
g
HDR (%)
Donor A
Donor B
Donor C
Donor D
Subpopulations

Supplementary Figure 4. *HBB*-gene targeted CD34⁺ HSPCs display improved long-term multi-lineage reconstitution in NSG mice. **(a)** Representative FACS plots showing gating scheme for analyses of NSG mice transplanted with human cells. Human engraftment was delineated as mCD45 negative and HLA-ABC positive. B cells were marked by CD19 expression, and myeloid cells identified by CD33 expression. **(b)** Human engraftment (16 weeks post-transplantation) in NSG mice from all experimental groups. Data from four bone marrow donors (donors A-D) represented in a separate panel. Median values reported + range reported. **(c)** Total HDR alleles in human cells in the bone marrow of NSG mice. Data from four bone marrow donors (donors A-D) represented in a separate panel. Median values + range reported. **(d)** Percentage of genome-edited cells that have successfully engrafted in the bone marrow of NSG mice. Calculated by % editing x % engraftment. Data from four bone marrow donors (donors A-D) represented in a separate panel. Median values reported + range reported. **(e)** Targeted allele frequency at *HBB* determined by ddPCR among engrafted human cells compared to targeting rate pretransplantation in an in vitro human HSPC population. Individual mice are represented. Median+interquantile range depicted. **f)** CD34⁺ HSPCs were sorted into sub-populations which were subsequently electroporated with Cas9-RNP and AAV6 with or without i53 peptide. HDR-mediated outcomes were assessed by ddPCR. Data from n=4 independent biological replicates with median±range depicted. (**g)** 4 bone marrow donors of CD34⁺ HSPCs were electroporated with Cas9-RNP and AAV6 with or without i53 peptide. HDR-mediated outcomes were assessed by ddPCR. Data from n=4 independent biological replicates.

Target	Sequence
HBB	CTTGCCCCACAGGGCAGTAA
CCR5	GCAGCATAGTGAGCCCAGAA
IL2RG	TGGTAATGATGGCTTCAACA
CFTR	TCTGTATCTATATTCATCAT
HBA1	CTACCGAGGCTCCAGCTTAA
TRAC	GAGAATCAAAATCGGTGAAT
AAVS1	AGTCCCACATGCTATCCACA
TET2	GCTCATGTGCAGTCACTGTG

Supplementary Table 1. sgRNA sequences. sgRNA sequences targeting various genomic locus

Template Name	Template Sequence
HBB HR	GTCCTGTAAGTATTTTGCA<br>GGAGACGCAGGAAGAGAT<br>ACATATCCCAAAGCTGAAT<br>gacaaaactcttccactttagtgcatca<br>gtgtaataagaaaattgggaaaacga<br>gcttaccaagctgtgaTTCCAAAT<br>TAAATACACTTGCAAAGGA<br>TTTTAGTAGCAATTTGTACT<br>TATGGGGCCAAGAGATATA<br>AGGGAGGGCTGAGGGTTT<br>CAACTCCTAAGCCAGTGCC<br>AGCCAAGGACAGGTACGG<br>TCACTTAGACCTCACCCTG<br>CACACCCTAGGGTTGGCC<br>CTCCCAGGAGCAGGGAGG<br>AGCCAGGGCTGGGCATAA<br>GGGCAGAGCCATCTATTGC<br>TTGCTTCTGACACAACTGT<br>TAGCAACCTCAAACAGACA<br>TGCACCTGACTCCTGAGGA<br>CGCAGTCACTGCCCTGTG<br>GGTGAACGTGGATGAAGTT<br>TGAGGCCCTGGGCAGGTT<br>AAGGTTACAAGACAGGTTT<br>GACCAATAGAAACTGGGCA<br>AGACAGAGAAGACTCTTG<br>GATAGGCACTGACTCTCTC<br>TTGGTCTATTTTCCCACCC<br>TGCTGGTGGTCTACCCTTG<br>[illegible]

Supplementary Table 2. HDR template sequence. HDR donor template encoding SCD correction upon targeting with sgRNA-Cas9 is listed.

Primer Name	Primer Sequence
HBB ddPCR-F	tcactagcaacctcaaacagac
HBB ddPCR-R	cctgtcttgtaaccttgatacc
HBB ddPCR- Reference - Probe	acgtggatgaagttggtggtgagg
HBB ddPCR - WT - Probe	ccccacagggcagtaacggcagacttc
HBB ddPCR- HR- Probe	tgactcctgaggaAaaAtcCgcAgtCa
IL2RG ddPCR-cDNA-F	GGGTGACCAAGTCAAGGAAG
IL2RG ddPCR-cDNA-R	GATGGTGGTATTCAAGCCGA
IL2RG ddPCR-cDNA-Probe	CAAGCGCCATGTTGAAACCCAGCCTGCCC
IL2RG ddPCR-Reference-F	GGGAAGGTAAAACTGGCAAC
IL2RG ddPCR-Reference-R	GGGCACATATACAGCTGTCT
IL2RG ddPCR Reference - Probe	CCTCGCCAGTCTCAACAGGGACCCAGC
HBA1 ddPCR- F	GCTGCCTATCAGAAAGTGGT
HBA1 ddPCR -R	TAGTGGGAACGATGGGGGAT
HBA1 ddPCR - probe	CTGGTGTGGCTAATGCCCTGGCCC
CCR5 ddPCR - F	GGGAGGATTGGGAAGACA
CCR5 ddPCR - R	AGGTGTTCAGGAGAAGGACA
CCR5 ddPCR - HR - Probe	GCTGGGGATGCGGTGG
CCR5 ddPCR - Reference - F	CCTCCTGGC GAGAAAAAG
CCR5 ddPCR - Reference - R	GTATGAATCCAGGTCC
CCR5 ddPCR - Reference - Probe	CAGGATAAGGCAGCTGT
HBB-AAVS1 translocation ddPCR - F	TCAGGGCAGAGCCATCTATTGC
HBB-AAVS1 translocation ddPCR - R	CCAGATAAGGAATCTGCCTAACAGG
5'–6FAM/ZEN/3'-IBFQ-labeled Probe (IDT)	CTTCTGACACAACTGTGTTCACTAGCAACC

Supplementary Table 3. ddPCR primers and probes. Primers and probes sequences used for ddPCR based assay are listed.

Primer Name	Primer Sequence
HBB out forward	aggaagcagaactctgcacttca
HBB in reverse	agtcagtgcctatcagaaacccaagag
HBB ICE forward	CCAACTCCTAAGCCAGTGCCAGAAGAG
HBB ICE reverse	AGTCAGTGCCTATCAGAAACCCAAGAG
CFTR forward	CCTTCTACTCAGTTTTAGTC
CFTR reverse	TGGGTAGTGTGAAGGGTTCAT
CFTR ICE primer	AGGCAAGTGAATCCTGAGCG
TET2 forward	CAAGCGGAATCCCATCTAAA
TET2 reverse	GGGTCTTGTTTCCTGCAAAA

Supplementary Table 4. Primers for ICE and TIDE analyses. Primers used for ICE and TIDE analyses are listed.
