# Supplementary material for: Transient inhibition of 53BP1 increases the frequency of targeted integration in human hematopoietic stem and progenitor cells
Source: Nat Commun. 2024 Jan 2;15:111. doi: 10.1038/s41467-023-43413-w (PMC10762240; doi:10.1038/s41467-023-43413-w)
Supplement: Supplementary file 3 — Reporting Summary [file 41467_2023_43413_MOESM3_ESM.pdf]

nature portfolio

Corresponding author(s): Matthew Porteus

Last updated by author(s): 10/12/2023

Reporting Summary

Nature Portfolio wishes to improve the reproducibility of the work that we publish. This form provides structure for consistency and transparency in reporting. For further information on Nature Portfolio policies, see our Editorial Policies and the Editorial Policy Checklist.

Statistics

For all statistical analyses, confirm that the following items are present in the figure legend, table legend, main text, or Methods section.

n/a	Confirmed	
☐	☒	The exact sample size (*n*) for each experimental group/condition, given as a discrete number and unit of measurement
☐	☒	A statement on whether measurements were taken from distinct samples or whether the same sample was measured repeatedly
☐	☒	The statistical test(s) used AND whether they are one- or two-sided<br>*Only common tests should be described solely by name; describe more complex techniques in the Methods section.*
☒	☐	A description of all covariates tested
☐	☒	A description of any assumptions or corrections, such as tests of normality and adjustment for multiple comparisons
☐	☒	A full description of the statistical parameters including central tendency (e.g. means) or other basic estimates (e.g. regression coefficient) AND variation (e.g. standard deviation) or associated estimates of uncertainty (e.g. confidence intervals)
☐	☒	For null hypothesis testing, the test statistic (e.g. F, t, r) with confidence intervals, effect sizes, degrees of freedom and P value noted<br>*Give P values as exact values whenever suitable.*
☒	☐	For Bayesian analysis, information on the choice of priors and Markov chain Monte Carlo settings
☒	☐	For hierarchical and complex designs, identification of the appropriate level for tests and full reporting of outcomes
☒	☐	Estimates of effect sizes (e.g. Cohen's *d*, Pearson's *r*), indicating how they were calculated

Our web collection on statistics for biologists contains articles on many of the points above.

Software and code

Policy information about availability of computer code

Data collection	BD FACS Diva software (v 8.0; BD Biosciences), QuantaSoft software (v.1.7; BioRad), ABI Prism 7900HT Sequence detection system, BD Accuri C6 Plus software, STEMvision (stem cell technologies), NovaSeq using 2x150bp pair-end sequencing, BioRad QX200 ddPCR machine, LiCOR biosciences, FACS ARIA II (BD BIosciences),
Data analysis	Prism 7 GraphPad Software, FlowJo (V.10.6.1, Flowjo LLC), TIDE (Tracking of Indels by Decomposition), ICE (Inference of CRISPR Edits),CAST-seq analysis (prev described: Turchiano G et al, 2021, Cell Stem Cell; Rhiel M et al, 2023, Front Genome Ed;), STEMvision (Stem cell technologies)

For manuscripts utilizing custom algorithms or software that are central to the research but not yet described in published literature, software must be made available to editors and reviewers. We strongly encourage code deposition in a community repository (e.g. GitHub). See the Nature Portfolio guidelines for submitting code & software for further information.

Data

Policy information about availability of data

All manuscripts must include a data availability statement. This statement should provide the following information, where applicable:

- Accession codes, unique identifiers, or web links for publicly available datasets
- A description of any restrictions on data availability
- For clinical datasets or third party data, please ensure that the statement adheres to our policy

No large datasets were generated as part of this work. All reagents and protocols will be made available to researchers. Source data are provided with this paper.

Human research participants

Policy information about studies involving human research participants and Sex and Gender in Research.

Reporting on sex and gender	*Use the terms sex (biological attribute) and gender (shaped by social and cultural circumstances) carefully in order to avoid confusing both terms. Indicate if findings apply to only one sex or gender; describe whether sex and gender were considered in study design whether sex and/or gender was determined based on self-reporting or assigned and methods used. Provide in the source data disaggregated sex and gender data where this information has been collected, and consent has been obtained for sharing of individual-level data; provide overall numbers in this Reporting Summary. Please state if this information has not been collected. Report sex- and gender-based analyses where performed, justify reasons for lack of sex- and gender-based analysis.*
Population characteristics	*Describe the covariate-relevant population characteristics of the human research participants (e.g. age, genotypic information, past and current diagnosis and treatment categories). If you filled out the behavioural & social sciences study design questions and have nothing to add here, write "See above."*
Recruitment	*Describe how participants were recruited. Outline any potential self-selection bias or other biases that may be present and how these are likely to impact results.*
Ethics oversight	*Identify the organization(s) that approved the study protocol.*

Note that full information on the approval of the study protocol must also be provided in the manuscript.

Field-specific reporting

Please select the one below that is the best fit for your research. If you are not sure, read the appropriate sections before making your selection.

☒ Life sciences ☐ Behavioural & social sciences ☐ Ecological, evolutionary & environmental sciences

For a reference copy of the document with all sections, see nature.com/documents/nr-reporting-summary-flat.pdf

Life sciences study design

All studies must disclose on these points even when the disclosure is negative.

Sample size	Sample sizes were chosen based on cell and animal availability or number of experimental or control groups needed to draw conclusions, but were not calculated prior to experiments. The resulting data were sufficient to show significance of reported data based on magnitudes of differences between groups.
Data exclusions	Any exclude data was either due to death of mouse or loss of animal organ prior to a complete analysis was carried out.
Replication	For all figures, multiple independent experiments were performed and all attempts at replicating observation as described in the manuscript were successful. Number of biological replicates varied between experiments and are detailed within the figure legend text.
Randomization	Recipient mice were randomly selected for transplantation groups. All mouse studies reported in this paper were performed as a minimum of three separate experimental replicates of editing and transplantation. For sample size, we transplanted as many mice as feasible to cover the non-Gaussian distribution that would be expected from experimental and donor variability, while also minimizing the total number of animals as per the FDA Center for Biologics Evaluation and Research guidelines.
Blinding	Blinding was not performed. Blinding was not performed as large number of animals and large number of experimental conditions were used for the study and we wanted to minimize complications from mixing up different experimental groups.

Reporting for specific materials, systems and methods

We require information from authors about some types of materials, experimental systems and methods used in many studies. Here, indicate whether each material, system or method listed is relevant to your study. If you are not sure if a list item applies to your research, read the appropriate section before selecting a response.

Materials & experimental systems

n/a	Involved in the study
☐	☒ Antibodies
☒	☐ Eukaryotic cell lines
☒	☐ Palaeontology and archaeology
☐	☒ Animals and other organisms
☒	☐ Clinical data
☒	☐ Dual use research of concern

Methods

n/a	Involved in the study
☒	☐ ChIP-seq
☐	☒ Flow cytometry
☒	☐ MRI-based neuroimaging

Antibodies

Antibodies used	Cells were stained with LIVE/DEAD Fixable Blue Dead Cell Stain (Life Technologies) and then with anti-human CD34 PE-Cy7 (581, BioLegend; 1:100), CD38 Alexa Fluor 647 (AT1, Santa Cruz Biotechnologies; 1;50), CD45RA BV 421 (HI100, BD Biosciences; 1:25), and CD90 BV605 (5E10, BioLegend; 1:30) and analysed by flow cytometry. For sorting of CD34+ or CD34+ CD38− CD90+ cells, cord-blood-derived CD34+ HSPCs were stained directly after isolation from blood with anti-human CD34 FITC (8G12, BD Biosciences; 1:100), CD90 PE (5E10, BD Biosciences; 1:50), CD38 APC (HIT2, BD Bioscience; 1:50), monoclonal anti-human CD33 BV421(1:50 dilution, 6 ul in 300 ul of MNCs pelleted in MACS buffer (1×PBS, 2% fetal bovine serum, 2 mM EDTA); anti-human HLA-ABC FITC (1:100 dilution; W6/32; BioLegend); anti-human CD19 PerCp-Cy5.5 (1:20 dilution; HIB19; BD Biosciences); anti-mouse CD45.1 PE-Cy7 (1:200 dilution; A20; eBiosciences); anti-human CD34 APC (1:50 dilution; 581; BioLegend);
Validation	All antibodies used in this study were validated by the manufacturer for flow cytometry. Validation statements can be found on the manufacturer websites using the Cat# detailed above (www.biolegend.com; www.thermofisher.com/us/en/home/lifescience/antibodies/ebioscience ; www.bdbiosciences.com)..To list a few please refer to: https://www.thermofisher.com/antibody/product/CD45-1-Antibody-clone-A20-Monoclonal/A18711; https://www.scbt.com/p/at1-antibody-c-18; https://www.biolegend.com/en-us/products/purified-anti-human-cd90-thy1-antibody-4110?GroupID=BLG5826. Please refer to the website for additional specific validation statements for each antibody as there are too many to list here

Animals and other research organisms

Policy information about studies involving animals; ARRIVE guidelines recommended for reporting animal research, and Sex and Gender in Research

Laboratory animals	6-8 weeks old NSG mice (male and female) were used for in vivo studies
Wild animals	Wild animals were not used
Reporting on sex	Male and Female mice were used in this study
Field-collected samples	study did not involved field data collection
Ethics oversight	All animal experiments were approved by the Administrative Panel on laboratory Animal Care at Stanford University

Note that full information on the approval of the study protocol must also be provided in the manuscript.

Flow Cytometry

Plots

Confirm that:

☒ The axis labels state the marker and fluorochrome used (e.g. CD4-FITC).

☒ The axis scales are clearly visible. Include numbers along axes only for bottom left plot of group (a 'group' is an analysis of identical markers).

☒ All plots are contour plots with outliers or pseudocolor plots.

☒ A numerical value for number of cells or percentage (with statistics) is provided.

Methodology

Sample preparation	Level of human engraftment assessed using total bone marrow. At week 16 or later, mouse bones were harvested from tibiae, femurs, sternum, and spinal cord from each mouse, and grinded using a mortar and pestle. Mononuclear cells (MNC) were purified using Ficoll gradient centrifugation (Ficoll-Paque Plus, GE Healthcare) for 25 mins at 2000g at room temperature. C. Red blood cells were lysed following a 10-12 min<br>incubation on ice with 500ul of 1x ACK lysis buffer (ThermoScientific, cat no. A1049201). Reaction was quenched and cells were<br>washed with MACS buffer (2% - 5% FBS, 2mM EDTA and 1x PBS). Peripheral blood samples were treated with 500 ul of 2% Dextren and incubated at 37C for 30 min to 1h. 800 ul to 1ml of the top layer was transferred to a FACS tube, spun down at 300<br>x g, 5 min and red blood cells lysed as already described. Cells purified from all 4 sources were re-suspended in 50 ul MACS buffer, blocked, stained with LIVE/Dead staining solution and stained for 30 min at 4°C, dark with pre-determined antibody panel, described in material and methods section.
Instrument	Accuri, BD FACS Aria II, Odyssey CLximager
Software	Accuri, BD FACS Aria II. All flow data are analyzed using Flowjo10
Cell population abundance	Detailed post-sort analysis is provided in the supplemental material
Gating strategy	A detailed gating strategy is provided in the supplemental material.

☒ Tick this box to confirm that a figure exemplifying the gating strategy is provided in the Supplementary Information.
